# West’s Lectures on the Diseases of Women

**Published:** 1859-01

**Authors:** 


ART. V.—*Lectures on the Diseases of Women.* By CHARLES WEST, M. D., Author of Lectures on the Diseases of Children, &c., &c. Part II. Philadelphia: BLANCHARD & LEA. 1858.

In the April number of this journal, we noticed the first part of this series of lectures, now completed in the present volume.

Dr. West has finished his work on the Diseases of Women much earlier than was anticipated from the announcement accompanying the first part. The volume before us contains the twelve concluding lectures of the series into which, for convenience, he has divided his book; for it is the design to include the two separate volumes into one, for which purpose the paging and numbering of the lectures are continuous with the first part, the two portions, when combined, forming a volume of five hundred pages.

Part second is devoted to the consideration of the diseases of the ovaries, vagina, bladder, and external organs.

The broad field of observation enjoyed by the author, in private and hospital practice, has been faithfully improved, and the profession at large are made his debtors, for the industry with which he has improved these opportunities for their benefit. We have already expressed, in our notice of the first part, an opinion of the value of this work on Female Diseases, and we should not repeat them if we should employ the ordinary language of complaint or commendation.

The work can scarcely, however, be regarded as a complete treatise upon *all* the ills of females, and a qualification in the title, expressive of the fact, might not be inappropriately made. A partial acknowledgment of this fact is made in the preface of the second part, in which Dr. West says: "A shorter time than I feared has sufficed for the fulfilment of my pledge, in the completion of this work. Many subjects, indeed, that deserve a longer notice, are touched on here but slightly, and others, of a purely surgical nature, are completely passed over, for I have not ventured to teach concerning matters, with reference to which I feel myself to be still altogether a learner, while I have always regarded mere compilation, uncontrolled by large experience, as more apt to peperpetuate error than to diffuse truth."

In fact, the great value of the book is in the originality of its matter. The diseases treated of are such as have come directly under his observation, and been made the subjects of careful study and analysis. The result is, that the several diseases treated of, and the number, as will be observed, is quite limited, are brought fully up to our present knowledge, upon all the points involved in their etiology, diagnosis, and treatment, But few persons exceed Dr. West in the perspicuity with which he states his facts, and arranges his figures, for the purpose of deducing a law in medicine; nor in the industry with which he collects every line of evidence having reference to the subject before him. Although we cannot, at all times, yield to the conclusions he draws, and are not willing to regard his judgment as infallible in all things, expressions of which dissent we have already indulged in, there is a freedom from dogmatism, in the manner he treats his subjects, which relieves any errors of judgment from offence in their declaration; and as he never fails to give the data from infallible statistics, from which he draws his own conclusions, he furnishes to his opponents the means of refuting his arguments; failing to do which, his dissenters are disarmed, in a great measure, from just causes of complaint, for any errors his teachings may inculcate.

The present volume contains fewer points for controversy than the first volume. The subjects differ in their character, and mooted points in pathology find little, or no space. The greater portion of the volume relates to subjects upon which the profession is content to sit quietly down, and patiently collect facts, progress, and results.

The greater portion of the volume is devoted to the consideration of the *uterine appendages*, a stereotyped term of medical phraseology, which he regards as exceptional, so far as it is employed in drawing diagnostic distinctions relative to the importance of the parts involved, but which he retains and employs, " merely as a convenient epithet, expressing, without waste of words, the broad ligaments of the uterus, and all the various parts and structures contained within, or intimately connected with them; parts whose physiological import just now concerns us less than do the ailments to which they are liable."

The first two lectures are devoted to the consideration of *inflammatory affections of the appendages*, which includes the affection of the ovaries themselves, and the cellular tissues in the immediate neighborhood of the uterus; and, *hæmorrhage* about the uterus, or *uterine hæmatocele.*

Their chief value, aside from the carefully drawn experience of the author, is the knowledge inculcated in drawing the nicely defined lines of diagnostic distinction between the precise parts involved. The sequelæ of the cases coming under the notice of Dr. West, prove that these troubles demand the most careful consideration of the physician during treatment, and that convalescence is not infrequently attended with complications, giving rise to anxiety, to both patient and attendant.

*Uterine hæmotocele*, or the formation of tumors in the immediate vicinity of the uterus, by the effusion of blood either into the cellular tissues around the womb, or into the peritoneal cavity, in the *cal-de-sac*, between the uterus and rectum, has attracted attention only within a few years, and chiefly by French writers. The bibliography of the subject is succinctly stated by Dr. West, and, we should be compelled to copy his entire note, if we attempted to embody here the historic notices of this affection.

This form of hæmorrhage is generally associated with some previous disorder of the menstrual function, often with its temporary suppression, the congestion of the sexual organs relieving itself by a proper outpouring of blood. Some of these cases have terminated fatally.

Four cases have come under the notice of Dr. West, and he finds accounts, more or less perfect, of thirty-seven cases in addition, eight of the whole number, or 19.5 per cent. proving fatal.

He remarks, that "we learn, then, from these observations, the existence of a previously unknown hazard, attendant on disorders of the sexual system in women; that not merely may intense congestion lead to profuse and dangerous floodings, or functional disturbance issue in inflammation of parts in the vicinity of the uterus, but also that vessels may gave way, and hæmorrhage take place inwardly, in situations where it is hard to discover, and still harder to suppress. As might be expected, the accident is one which takes place only during the period of sexual vigor, it having occurred in twenty-one women at the following ages:

| | |
|---|---|
| Under 20, in . . . . . . . | 2 |
| Between 20 and 25, in . . . . . | 2 |
| " 25 " 30 " . . . . . . | 7 |
| " 30 " 35 " . . . . . | 5 |
| " 35 " 40 " . . . . . | 4 |
| At 40, in . . . . . . . . . | 1 |
| | 21 |

Of the above 21 patients, 15 were married, 3 were single, and the civil state of the other 3 is not mentioned."

We have room only for one other remark of Dr. West in reference to this subject:

"There are *four conditions* with this *uterine hæmatocele may be confounded*, viz., extra-uterine pregnancy, retroversion of the pregnant uterus, inflammation of the cellular tissue, between the uterus and rectum, and ovarian tumor; and the points of similarity between each of these are quite sufficient to lead very readily into error."

The whole subject is suggestive of the rapidly advancing progress of differential diagnois upon every point in the wide domain of medicine, and suggests farther, that vigilance, like the price of liberty, is the only coin with which can be purchased positive advancement in our profession, and secure to its followers honorable distinction.

The twenty-third lecture is occupied with the consideratiou of inflammatory affections of ihe ovaries, acute and chronic. The symptoms are traced with faithfulness, and we shall forbear to question the correctness of some of his conclusions, which locate in one of these degrees of inflammation of the ovaries, the sexual derangements present, instead of making the ovaries secondary in the chain of symptoms, and giving to the uterine neck the primary place in the sequence of cause and effect. While the subject is in its present unsettled state, almost every practitioner has, notwithstanding the influence of great names arrayed upon either side, made up his own mind in reference to the matter, based upon his own observation and experience, and formed settled opinions upon the questions involved in dispute. At the same time, the physician would be unpardonable for permitting his preconceived opinions to blind him to the possibility of the primary seat of these difficulties, having a place in either organs. To learn to reason accurately, and judge correctly, is the great duty of the physician; and happy is he, if he can plead that his errors are the errors *only* of an *unbiassed* judgment.

The six succeeding lectures, which, it will be observed, constitute one-half of the volume, are occupied with the consideration of *ovarian tumors and dropsy.*

It is, perhaps, sufficient to say, that the subject is treated in an able manner. Every fact bearing upon the subject, has been laboriously collected, and presented in the most lucid light. The author's own knowledge of the subject is drawn from the observation of sixty-eight cases. He has added such reference to the contributions of others, as to render the lectures a complete epitome of the subject, and it would be useless for us to endeavor to compress into our present notice, the statistics which he has collected in reference to the various points involved in the consideration of the disease. It is gratifying to notice the justice done to American surgery in the treatment of the subject.

The space occupied in the volume is such as, perhaps, to seem to demand an extended notice. But we cannot, after a careful perusal of every page, find the point, or points, which will "adorn" a review. The analytical powers of the author are preëminently brought into play in these sections; what he has learned from others is displayed with all the perspicuity which figures afford him, and what his own experience teaches him, is modestly stated, and which only an equal experience qualifies one to question. We can only simply recommend these lectures to the study of those desiring to perfect themselves in this branch of female therapeutics.

In reference to *treatment*, basing his conclusions upon the results of his own sixty-eight cases, and a careful examination of numerous recorded cases, he much prefers tapping, and the injection of iodine into the cyst, to extirpation of the tumor, or tumors. In reference to the results of ovariotomy, he has collected the statistics, with an evident intention of fairness, which will render it difficult, unless new figures are presented, to refute; the results of which figures are, that "of 292 recorded instances of the operation being attempted, 120 ended in death, and 92 in failure; or, in other words, the chances are two to one that the operation will be accomplished; but, if it succeeds, they are nearly equal, that the patient will die; and if it fails, the prospect of her surviving the fruitless interference is only double that of her sinking in consequence of it." Nor does he think, the statement of reporters to the contrary, that the rate of mortality is decreasing, from our increased skill in diagnosis and operating.

The succeeding and concluding three lectures are given to the considera-tion of *diseases of the female bladder*, *of the urethra and vagina, and external organs of generation.*

These subjects are all treated with the same skill which marks the pre-ceding lectures, and add to our stock of medical lore; but we shall answer the purposes we designed in the present notice, by a simple reference to their contents.

Forming, as this volume does, only the conclusion of the previous part, we shall apply the general terms of commendation, employed in our notice of the first portion of the work, to these concluding lectures, in order to avoid the repitition of the language then used. We presume but few med-ical libraries will be long found without the work entire, as the well known reputation of the author will furnish a speedy passport to the notice of the profession, irrespective of critical praise, or censure. N.

